# Incidence, risk factors, and clinical impact of major bleeding in hospitalized patients with COVID-19: a sub-analysis of the CLOT-COVID Study

**DOI:** 10.1186/s12959-022-00414-x

**Published:** 2022-09-20

**Authors:** Junichi Nakamura, Ichizo Tsujino, Sen Yachi, Makoto Takeyama, Yuji Nishimoto, Satoshi Konno, Naoto Yamamoto, Hiroko Nakata, Satoshi Ikeda, Michihisa Umetsu, Shizu Aikawa, Hiroya Hayashi, Hirono Satokawa, Yoshinori Okuno, Eriko Iwata, Yoshito Ogihara, Nobutaka Ikeda, Akane Kondo, Takehisa Iwai, Norikazu Yamada, Tomohiro Ogawa, Takao Kobayashi, Makoto Mo, Yugo Yamashita, the CLOT-COVID Study Investigators

**Affiliations:** 1 Division of Respiratory and Cardiovascular Innovative Research, Faculty of Medicine, Hokkaido University, North 14, West 5, Kita-ku, Sapporo, 060-8648 Japan; 2 Hokkaido University Hospital, Sapporo, Japan; 3 Japan Community Health Care Organization Tokyo Shinjuku Medical Center, Tokyo, Japan; 4 Hyogo Prefectural Amagasaki General Medical Center, Amagasaki, Japan; 5 Hamamatsu Medical Center, Hamamatsu, Japan; 6 Yokosuka General Hospital Uwamachi, Yokosuka, Japan; 7 Nagasaki University Graduate School of Biomedical Sciences, Nagasaki, Japan; 8 Tohoku University Hospital, Sendai, Japan; 9 Tsukuba Medical Center Hospital, Tsukuba, Japan; 10 Osaka Metropolitan University Graduate School of Medicine, Osaka, Japan; 11 Fukushima Red Cross Hospital, Fukushima, Japan; 12 Kyoto University Hospital, Kyoto, Japan; 13 Nankai Medical Center Japan Community Health Care Organization, Saiki, Japan; 14 Mie University Hospital, Tsu, Japan; 15 Toho University Ohashi Medical Center, Tokyo, Japan; 16 Shikoku Medical Center for Children and Adults, Zentsuji, Japan; 17 Tsukuba Vascular Center, Ibaraki, Japan; 18 Kuwana City Medical Center, Kuwana, Japan; 19 Fukushima Daiich Hospital, Fukushima, Japan; 20 Yokohama Minami Kyosai Hospital, Yokohama, Japan

**Keywords:** COVID-19, Bleeding, Severity, Anticoagulant, Hospitalization, Mortality

## Abstract

**Background:**

The coronavirus disease 2019 (COVID-19) causes extensive coagulopathy and a potential benefit of anticoagulation therapy has been documented for prevention of thromboembolic events. Bleeding events has also been reported as a notable complication; whereas, the incidence, risks, and clinical impact of bleeding remain unclear.

**Method:**

The CLOT-COVID Study was a nationwide, retrospective, multicenter cohort study on consecutive hospitalized patients with COVID-19 in Japan between April 2021 and September 2021. In this sub-analysis, we compared the characteristics of patients with and without major bleeding; moreover, we examined the risk factors for and clinical impact of bleeding events.

**Results:**

Among 2882 patients with COVID-19, 57 (2.0%) had major bleeding. The incidence of major bleeding increased with COVID-19 severity as follows: 0.5%, 2.3%, and 12.3% in patients with mild, moderate, and severe COVID-19, respectively. COVID-19 severity, history of major bleeding, and anticoagulant type/dose were independently and additively associated with the bleeding incidence. Compared with patients without major bleeding, those with major bleeding exhibited a longer duration of hospitalization (9 [6–14] vs 28 [19–43] days, *P* < 0.001) and higher mortality during hospitalization (4.9% vs. 35.1%, *P* < 0.001).

**Conclusions:**

In the real-world clinical practice, the incidence of major bleeding was not uncommon, especially in patients with severe COVID-19. Independent risk factors for major bleeding included history of major bleeding, COVID-19 severity, and anticoagulant use, which could be associated with poor clinical outcomes including higher mortality. Precise recognition of the risks for bleeding may be helpful for an optimal use of anticoagulants and for better outcomes in patients with COVID-19.

## Introduction

The coronavirus disease 2019 (COVID-19) causes unique and extensive coagulopathy [1], with a high incidence of thromboembolic events reported especially in the lungs [2–5]. Thus, several current guidelines recommend that hospitalized patients with COVID-19 receive anticoagulation therapy for the prevention of thrombosis [6, 7]. Alternatively, prior studies have also reported a high incidence of bleeding complications among patients with COVID-19, especially those receiving a more intensive dose of anticoagulant [3, 8–15].

Identification of patients at increased risk for bleeding during anticoagulation therapy is clinically relevant in determining the optimal management strategies of anticoagulation therapy. However, there remain limited real-world data regarding the bleeding events for patients with COVID-19. In addition, impact of major bleeding on the duration of hospitalization and death in COVID-19 has been scarcely reported.

In the present study, we aimed to identify risk factors of major bleeding and identify the impact of major bleeding on clinical outcomes in hospitalized patients with COVID-19 by using a large-scale multicenter observational database of patients with COVID-19 in Japan. The findings of this study could inform optimal anticoagulant use and improve clinical outcomes in patients with COVID-19.

## Methods

### Study population

The CLOT-COVID Study (thrombosis and antiCoaguLatiOn Therapy in patients with COVID-19 in Japan Study: UMIN000045800) is a physician-initiated, retrospective, multicenter cohort study on 2894 consecutive patients hospitalized with COVID-19 in 16 Japanese centers between April 2021 and September 2021. The design of the study was previously reported in detail [16, 17]. The present study was performed by dedicated members of the Japanese Task Force for Venous Thromboembolism (VTE) and COVID-19 in Japan in a collaborative effort with the Japanese Society of Phlebology and the Japanese Society of Pulmonary Embolism Research. Using the hospital databases, we included consecutive patients diagnosed with COVID-19 via a polymerase chain reaction test.

### Ethics approval and consent to participate

All procedures were conducted following the Declaration of Helsinki. The research protocol was approved by the relevant review boards or ethics committees of all participating centers. The requirement of written informed consent was waived since we used clinical information obtained in routine clinical practice. This study protocol was in accordance with the guidelines for epidemiological studies issued by the Ministry of Health, Labor, and Welfare in Japan.

### Data collection

Patients’ data and follow-up information were collected using an electronic report form. Data regarding the patient characteristics, pharmacological thromboprophylaxis management, and clinical outcomes were collected from the hospital charts or databases based on pre-specified definitions. Data entry into electronic case report forms was performed by physicians at each institution. Furthermore, the integrity of the data was manually checked at the general office.

### Definitions for patient characteristics

Details of the definitions for the diagnosis of complications have been described in our prior publications [16, 17]. In brief, hypertension was defined as peripheral blood pressure > 140/90 mmHg or the use of medication for hypertension. Diabetes was diagnosed using hemoglobin A1c (HbA1c) or by the use of medication for diabetes. Heart disease was defined as heart disorders including heart failure and history of myocardial infarction. Respiratory disease was defined as persistent lung disorders including chronic obstructive pulmonary disease and restrictive lung diseases. Patients with active cancer were defined as individuals receiving cancer treatment; individuals scheduled to undergo cancer surgery; individuals with metastasis to other organs; and/or individuals with terminal cancer [18]. Patients with mild, moderate, and severe COVID-19 were defined as those who did not require oxygen supplementation, those who require oxygen supplementation, and those who require mechanical ventilation or extracorporeal membrane oxygenation, respectively.

Pharmacological thromboprophylaxis was evaluated by the usage of any anticoagulants during the hospitalization except for their usage for the treatment of thrombosis. An unfractionated therapeutic heparin dose was defined as the administration of unfractionated heparin targeting a therapeutic range with reference to the activated partial thromboplastin time (APTT). An unfractionated prophylactic heparin dose was defined as the administration of a fixed dose of unfractionated heparin without reference to the APTT. Anticoagulant use was classified into 4 groups as follows: no anticoagulant, parenteral prophylactic dose (low-molecular-weight heparin [LMWH] or unfractionated heparin [UFH]), oral therapeutic dose (warfarin or direct oral anticoagulant [DOAC]), and parenteral therapeutic dose (UFH). In Japan, administration of a therapeutic LMWH dose is not allowed; therefore, only UFH is administered as a parenteral therapeutic dose. We considered a therapeutic dose of warfarin or DOAC as a single group since patients receiving DOAC have a lower incidence of bleeding than those receiving parenteral therapeutic anticoagulants [12, 19].

### Clinical outcomes

The outcome measure in the current study was major bleeding during hospitalization, which was diagnosed based on the International Society of Thrombosis and Hemostasis (ISTH) criteria, which included a reduction in the hemoglobin level by ≥ 2 g/dL, transfusion of ≥ 2 units of blood, or symptomatic bleeding in a critical body region or organ [20].

### Statistical analysis

Categorical variables are expressed as numbers and percentages. Continuous variables are expressed as the mean and standard deviation or the median and interquartile range (IQR) based on the normality of distribution. Between-group comparisons of categorical variables were performed using the chi-square test, as appropriate; otherwise, Fisher’s exact test was used. Continuous variables were compared using Student's t-test or Wilcoxon’s rank-sum test based on the normality of distribution.

The crude odds ratio (OR) for major bleeding was calculated using univariate analyses of baseline characteristics. Based on previous reports [3, 8–10, 12–14, 21, 22] and clinical relevance, we selected 5 baseline characteristics, namely, age, sex, history of major bleeding, and severity of COVID-19 at admission, and pharmacological thromboprophylaxis. Subsequently, we estimated the adjusted OR and their 95% confidence interval (CI) after a constructing the multivariable logistic regression model excluding patients with missing thrombophylactic regimens during admission.

Regarding outcome analysis, we compared the hospitalization duration and mortality rate between patients with and without major bleeding. To adjust for possible confounding factors, we constructed multivariable logistic regression models that comprised age, sex, comorbid diseases (hypertension, diabetes mellitus, and active cancer), history of major bleeding, body mass index > 30 kg/m^2^, COVID-19 severity at admission, anticoagulation regiments, and VTE development during hospitalization based on clinical relevance and previous studies [23, 24]. We then examined whether the major bleeding independently affected the hospitalization duration and mortality.

All statistical analyses were performed using JMP Pro 15.0.0 (SAS Institute Inc., Cary, NC, USA). All reported P-values were 2-tailed. Statistical significance was set at a *P*-value < 0.05.

## Results

**Fig. 1:**
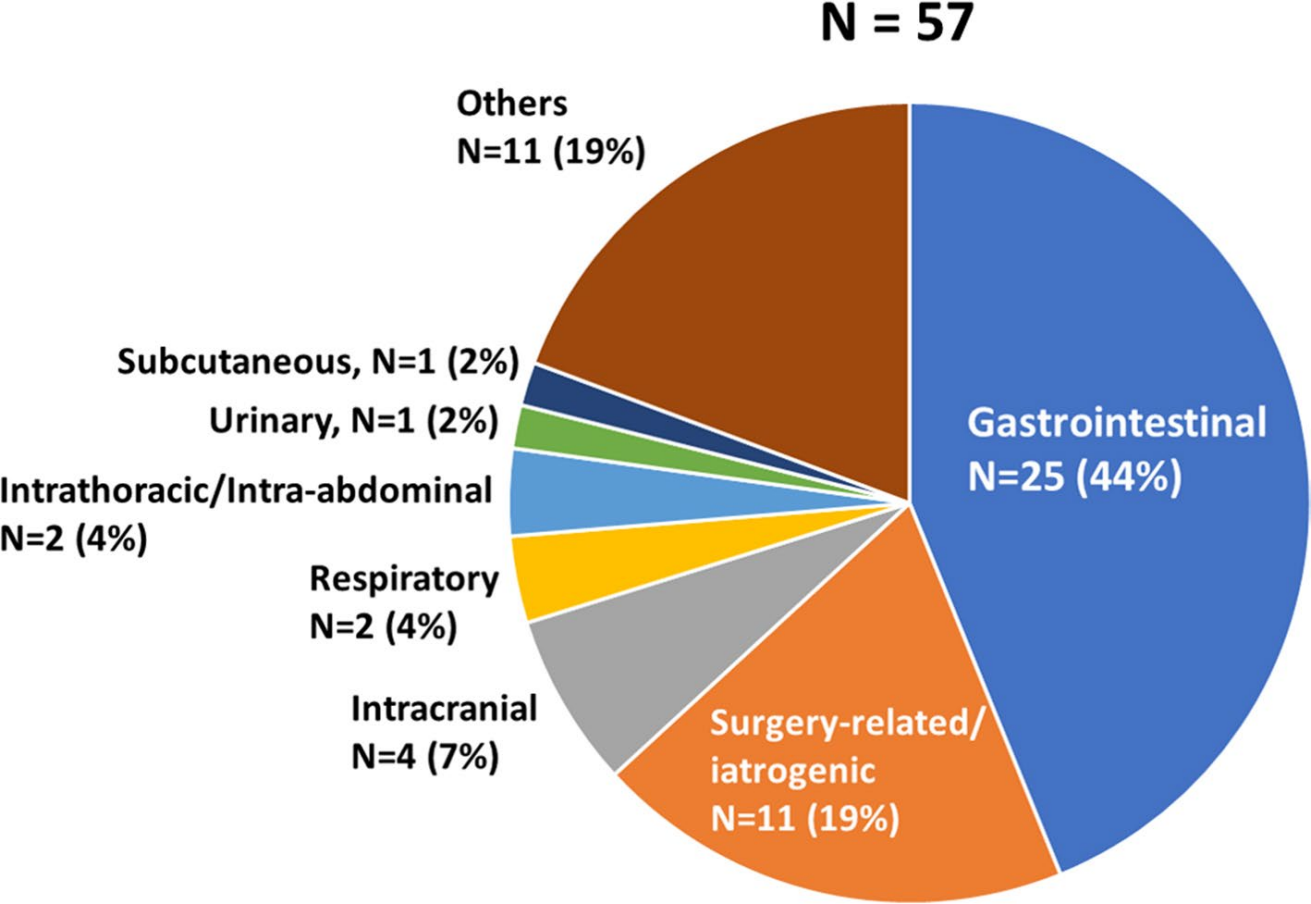
Sites of major bleeding. The bleeding sites in the 57 major bleeding events are shown

**Fig. 2:**
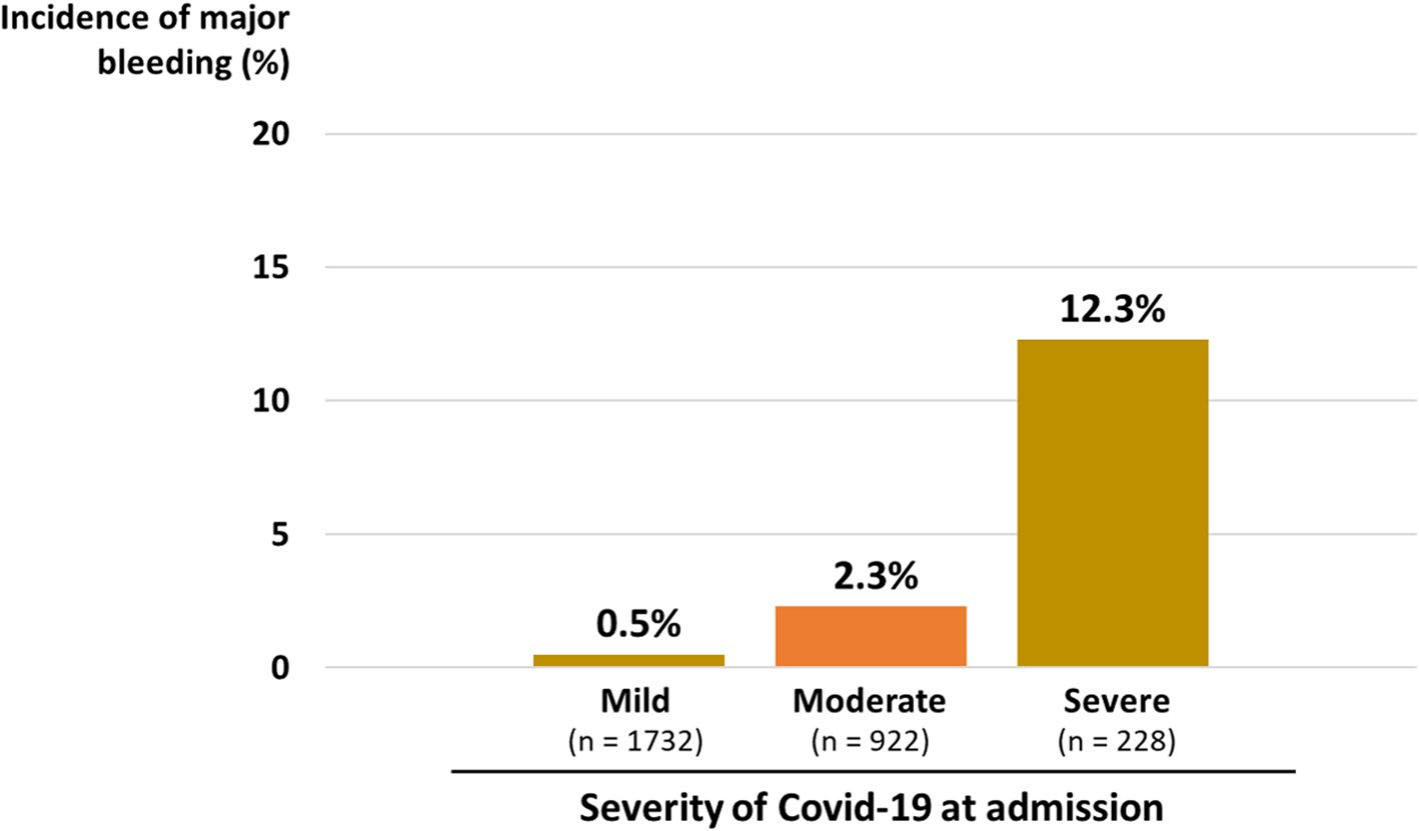
Number of major bleeding events according to COVID-19 severity. The proportion of patients with major bleeding among patients with mild, moderate, and severe COVID-19 is shown COVID-19, coronavirus disease 2019

Among 2,894 patients, 12 had incomplete data regarding the use of anticoagulants; accordingly, we analyzed data from the remaining 2,882 patients. Among them, 57 (2.0%) experienced major bleeding events. The gastrointestinal (GI) tract was the most common bleeding site (25/57; 44%), followed by surgery-related/iatrogenic (11/57; 19%) and intracranial bleeding (4/2,882; 7%) (Fig. 1). The incidence of major bleeding was 0.5% (8/1732), 2.3% (21/922), and 12.3% (28/228) among patients with mild, moderate, and severe COVID-19, respectively (Fig. 2). As shown in Table 1, there were significant differences in age, comorbidities (hypertension, diabetes mellitus, and heart disease), history of major bleeding, COVID-19 severity on admission, worst COVID-19 severity during hospitalization, and pharmacological thromboprophylaxis regimens between patients with and without major bleeding.

**Table 1:** Comparison of patient characteristics between patients with and without major bleeding

	**Total** **(*N* = 2882)**	**Patients with major bleeding** **(*N* = 57)**	**Patients without major bleeding** **(*N* = 2825)**	***P* value**
**Baseline characteristics**				
Age (years)	52.7 ± 17.9	61.7 ± 14.6	52.5 ± 17.9	<0.001
Men	1877 (65.1%)	42 (73.7%)	1835 (65.0%)	0.17
Body weight (kg)	68.8 ± 18.4	70.1 ± 14.4	68.8 ± 18.5	0.62
Height (cm)	164.3 ± 12.4	164.1 ± 8.7	164.3 ± 12.4	0.88
Body mass index (kg/m^2^)	25.3 ± 5.4	25.9 ± 4.6	25.2 ± 5.4	0.39
Body mass index > 30 kg/m^2^	456 (15.8%)	11 (19.3%)	445 (15.8%)	0.47
D-dimer level at admission (μg/mL)	0.8 (0.5–1.3)	1.6 (0.9–4.3)	0.8 (0.5–1.3)	0.05
**Comorbidities**				
Hypertension	869 (30.2%)	33 (57.9%)	836 (29.6%)	<0.001
Diabetes mellitus	595 (20.6%)	21 (36.8%)	574 (20.3%)	0.002
Heart disease	254 (8.8%)	12 (21.1%)	242 (8.6%)	0.001
Respiratory disease	298 (10.3%)	9 (15.8%)	289 (10.2%)	0.17
Active cancer	60 (2.1%)	2 (3.5%)	58 (2.1%)	0.45
History of major bleeding	26 (0.9%)	5 (8.8%)	21 (0.7%)	<0.001
History of VTE	15 (0.5%)	0 (0%)	15 (0.5%)	0.58
**Severity of COVID-19 at admission**				
Mild	1732 (60.1%)	8 (14.0%)	1724 (61.0%)	<0.001
Moderate (Need oxygen)	922 (32.0%)	21 (36.8%)	901 (31.9%)	
Severe (Need mechanical ventilation or ECMO)	228 (7.9%)	28 (49.1%)	200 (7.1%)	
**Worst severity of COVID-19 during hospitalization**				
Mild	1278 (44.3%)	4 (7.0%)	1274 (45.1%)	<0.001
Moderate (Need oxygen)	1225 (42.5%)	14 (24.6%)	1211 (42.9%)	
Severe (Need mechanical ventilation or ECMO)	379 (13.2%)	39 (68.4%)	340 (12.0%)	
**Pharmacological thromboprophylaxis regimens**				
Non-anticoagulants	1649 (57.2%)	6 (10.5%)	1643 (58.2%)	<0.001
Anticoagulants	1233 (42.8%)	51 (89.5%)	1182 (41.8%)	
Prophylactic dose (LMWH or UFH)	889/1233 (72.1%)	20/51 (39.2%)	869/1182 (73.5%)	-
Therapeutic dose (UFH)	161/1233 (13.1%)	26/51 (51.0%)	135/1182 (11.4%)	-
Therapeutic dose (Warfarin or DOAC)	183/1233 (14.8%)	5/51 (9.8%)	178/1182 (15.1%)	-

LMWH was used with a prophylactic dose alone since its use as a therapeutic dose is not allowed in Japan. Regarding UFH, its prophylactic dose was defined as the administration of a fixed dose without reference to the APTT while the therapeutic dose was defined as administration of a therapeutic dose with reference to the APTT

*VTE* Venous thromboembolism, *COVID-19*, Coronavirus disease 2019, *ECMO* Extracorporeal membrane oxygenation, *APTT* Activated partial thromboplastin time, *CT* Computed tomography, *LMWH* Low molecular weight heparin, *UFH* Unfractionated heparin, *DOAC* Direct oral anticoagulant

As shown in Table 2, multivariate analysis revealed significant between-group differences in the history of major bleeding, COVID-19 severity at admission, and pharmacological thromboprophylaxis.

**Table 2:** Univariate and multivariable analyses for the risk of major bleeding

	**Univariate analysis**		**Multivariable analysis**	
	**Crude OR** **(95% CI)**	**P-value**	**Adjusted OR** **(95% CI)**	***P*-value**
Age (per 1 year)	1.03 (1.01–1.05)	< 0.001	1.01 (0.99–1.04)	0.17
Men	0.66 (0.37–1.20)	0.17	1.20 (0.63–2.29)	0.58
History of major bleeding	12.8 (4.66–35.7)	< 0.001	10.8 (3.16–36.6)	< 0.001
**Severity of COVID-19 at admission**				
Mild (Reference)	–	–	–	
Moderate	5.02 (2.22–11.4)	< 0.001	1.98 (0.77–5.13)	0.16
Severe	30.2 (13.6–67.1)	< 0.001	6.15 (2.24–16.9)	0.001
**Pharmacological thromboprophylaxis**				
No anticoagulants (Reference)	–	–	–	
Prophylactic dose (LMWH or UFH)	6.30 (2.52–15.8)	< 0.001	3.02 (1.04–8.82)	0.04
Therapeutic dose (Warfarin or DOAC)	7.69 (2.32–25.5)	< 0.001	3.17 (0.84–12.0)	0.09
Therapeutic dose (UFH)	52.7 (21.3–130.3)	< 0.001	13.7 (4.27–44.2)	< 0.001

Age, sex, history of major bleeding, severity of COVID-19 at admission, and pharmacological thromboprophylaxis were included in the multivariate analysis

*OR* Odds ratio, *CI* Confidence interval, *COVID-19* Coronavirus disease 2019, *LMWH* Low molecular weight heparin, *UFH* Unfractionated heparin, *DOAC* Direct oral anticoagulant, *vs* Versus

Here, regarding the adjusted OR for major bleeding, it was 1.98 (CI 0.77–5.13) for moderate COVID-19 as compared with the mild COVID-19 group, whereas it was even higher (OR 6.15 [CI 2.24–16.9]) for severe COVID-19. Similarly, the adjusted OR for major bleeding was approximately 3 for both groups with a prophylactic dose of parenteral anticoagulant (OR 3.02 [CI 1.04–8.82]) and a therapeutic dose of warfarin/DOAC (OR 3.17 [CI 0.84–12.0]), whereas it was even higher (13.7 [CI 4.27–44.2]) for patients with a therapeutic dose of UFH. Based on these stepwise results, we made a preliminary scoring system in which the risk was assessed in each patient by adding the risk points described below:

- History of major bleeding: no, 0 points; yes, 1 point
- COVID-19 at admission: mild, 0 points; moderate, 1 point; severe, 2 points
- Anticoagulant regimen: no anticoagulants, 0 points; a prophylactic dose of parenteral anticoagulant (UFH or LMWH) or therapeutic dose of warfarin/DOAC, 1 point; a therapeutic dose of UFH, 2 points

**Fig. 3:**
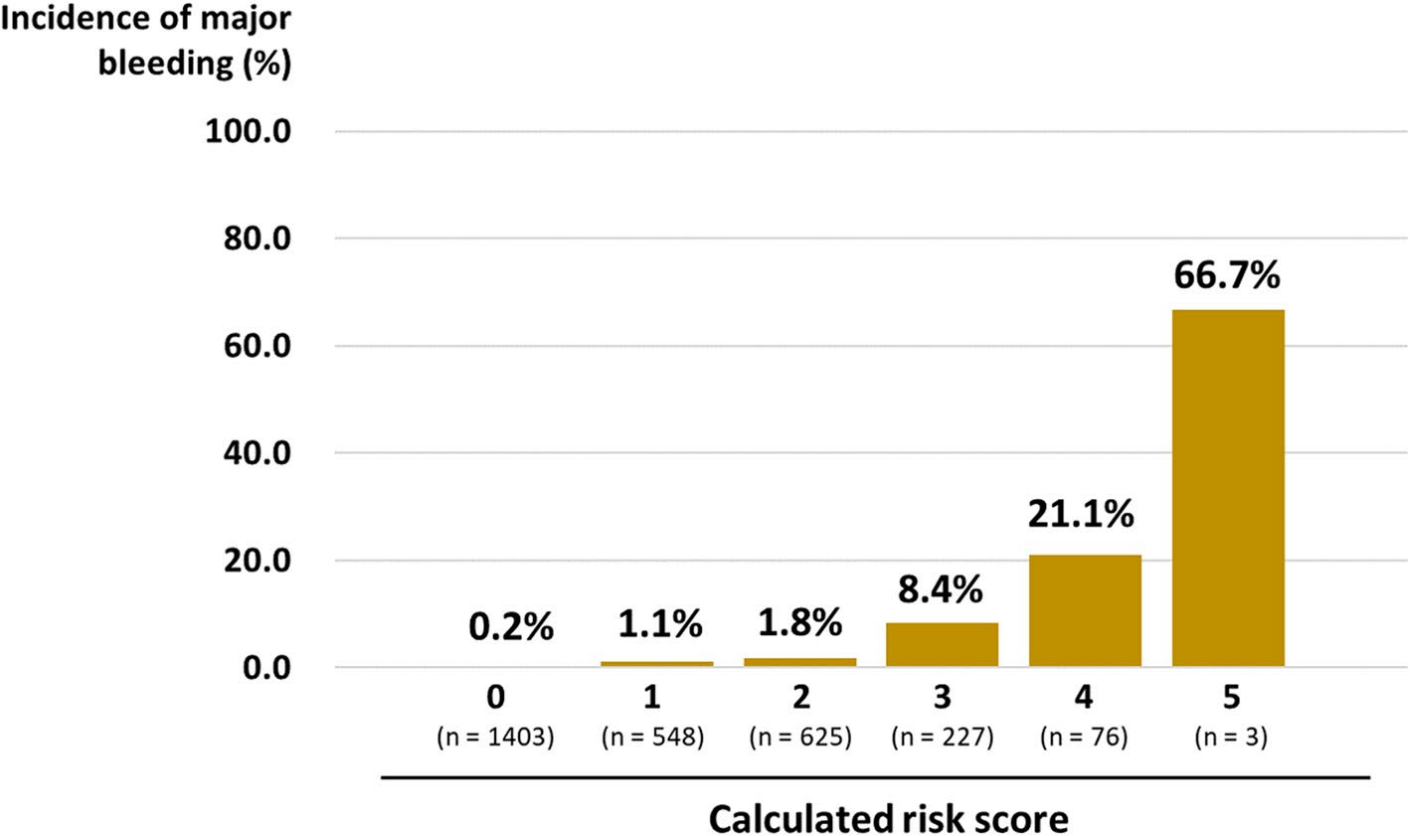
Incidence of major bleeding according to the calculated risk score. The proportion of patients with major bleeding according to the total risk score of major bleeding is shown. The risk score was calculated by summing the points described below. History of major bleeding: no, 0 point; yes, 1 point. COVID-19 severity at admission: mild, 0 point; moderate, 1 point; severe, 2 points. Anticoagulant regimen: no anticoagulants, 0 point; prophylactic dose of parenteral anticoagulant (unfractionated heparin or low molecular weight heparin) or therapeutic dose of warfarin/DOAC, 1 point; therapeutic dose of unfractionated heparin, 2 points. COVID-19, coronavirus disease 2019; LMWH, low-molecular-weight heparin; UFH, unfractionated heparin; DOAC, direct oral anticoagulant

Figure 3 shows the proportion of patients with major bleeding based on the calculated risk score, where the incidence of bleeding was positively correlated with the calculated score.

Patients with major bleeding had a significantly longer duration of hospitalization than those without major bleeding (28 [19–43] vs 9 [6–14] days, *P* < 0.001), and this difference remained significant in the multiple logistic regression analysis (*P* < 0.001). Similarly, the mortality rate was significantly higher among patients with major bleeding (20/57, 35.1%) compared to those without major bleeding (138/2825, 4.9%) (*P* = 0.02). In the multiple logistic regression analysis, the adjusted OR and 95% CI for major bleeding for death were 2.27 and 1.10–4.93, respectively.

## Discussion

Our main findings were as follows: 1) the overall incidence of major bleeding was 2.0% (57/2894), which reached as high as 12.3% in patients with severe COVID-19; 2) the GI tract was the most common bleeding site; 3) the incidence of major bleeding was independently associated with history of major bleeding, COVID-19 severity at admission, and anticoagulant use; 4) the incidence of major bleeding was positively correlated with the accumulated risks; and 5) major bleeding was an independent risk factor for longer duration of hospitalization and higher mortality.

The reported incidence of major bleeding in hospitalized patients with COVID-19 ranges from 0.5 to 11.4% in prior studies [3, 8–14, 25, 26], which varies depending on the COVID-19 severity, anticoagulation drugs, and geographical differences in the availability of medical resources. In our study enrolling consecutive COVID-19 patients with any disease severities, the overall incidence of major bleeding was 2.0%, which was consistent with previous reports. However, the incidence rate increased to 12.3% among patients with severe COVID-19, which is higher than previously reported incidence rates in severe status of COVID-19 (3.0%–10.6%) [3, 8, 10, 14]. This indicates a critical need for an attention against bleeding events when managing patients with severe COVID-19. Notably, the incidence of major bleeding is not significantly higher in COVID-19 than in other critical illnesses [3, 27]. This is intriguing because thrombotic events, especially pulmonary thromboembolism, have a higher incidence rate among patients with COVID-19 than in those with other acute illnesses [28]. This could be attributed to a COVID-19-specific coagulopathy characterized by in-situ formation of immune-thrombosis in the lungs [1]. Further research on this is required.

In our study, the most common bleeding site was the GI tract, followed by surgery-related/iatrogenic and intracranial bleeding, which is consistent with previous reports on patients with COVID-19 [27, 29–32] and other patients on anticoagulants [33, 34]. This suggests that COVID-19 lacks a disease-specific profile for bleeding sites. Gastric ulcers are the most common cause of GI tract bleeding in patients with COVID-19 [35], which suggests that preventive medication, such as proton pump inhibitors, is especially important in COVID-19. This is because diagnostic workup, including endoscopy, is substantially limited given the high transmissibility of the virus. Contrastingly, in our study, intracranial bleeding developed only in a small number of patients (4 cases), which is consistent with previous reports [12, 36]. Nevertheless, intracranial bleeding tends to develop in young individuals and can be fatal in patients with COVID-19 [37]. Therefore, the possibility of intracranial bleeding should be carefully considered when handling patients with COVID-19.

Previous studies reported that risk factors for major bleeding in COVID-19 included high levels of D-dimer and ferritin, COVID-19 severity, and anticoagulant use [8, 9, 15, 29, 31, 36, 38]; among these, high-dose anticoagulant administration has been consistently associated with an increased risk of major bleeding. This is consistent with our findings, where patients receiving a therapeutic dose of anticoagulants showed a markedly higher risk for major bleeding compared with those without anticoagulation. These findings demonstrate the importance of an optimal use of anticoagulants in patients with COVID-19. Specifically, the administration of a therapeutic dose of anticoagulants should be carefully considered especially in patients with mild COVID-19 given their low risk of VTE [39]. Alternatively, as recommended by Kessler et al., de-escalating the anticoagulant dose may be considered upon the improvement of the COVID-19 severity and reduction of the risk of pulmonary thromboembolism [38].

A recent Spanish study reported an increased bleeding risk among patients with multiple risk factors [8]. Further, they proposed a grading system that included intensive care unit stay, D-dimer and ferritin levels, and therapeutic anticoagulation [9]. Here, high-risk and very-low-risk patients had a incidence of major bleeding of 15.4% and 1%, respectively. Consistent with these previous reports, we observed that the incidence rate of major bleeding was positively correlated with the accumulated risks. This suggests the importance of evaluating multiple bleeding-related risk factors when handling patients with COVID-19. However, risk factors related to potential bleeding could vary according to disease severity, ethnicity, and geographical areas. Accordingly, further research is warranted to establish a method for an easy and precise estimation of the risk of bleeding in patients with COVID19.

Among patients with COVID-19, those with bleeding events have a higher mortality rate than those without [8, 40–42]. Accordingly, we observed that major bleeding was independently associated with higher mortality. Further, patients with major bleeding had a longer duration of hospital stay than those without. Sex, hospital location, and pre-existing kidney or liver disease are factors that affect the hospitalization duration [23, 43]; however, it remains unclear how bleeding events affect the duration of hospitalization in patients with COVID-19. Given the considerable impact of the hospitalization duration on the cost and burden to the medical staff and facilities, further research is warranted on the effect of in-hospital events, including bleeding, on the hospitalization duration.

This study has several limitations. First, there was a small number of bleeding events; therefore, we only included a limited number of variables in the multivariable analysis. Second, this was a retrospective observational study, which could result in various biases. For example, the therapeutic decision-making, including pharmacological thromboprophylaxis, was left to the discretion of the attending physicians, which could have affected clinical outcomes such as death and hospitalization duration. Finally, we did not examine blood parameters, including serum ferritin, which are associated with major bleeding [8, 9, 25, 41]. However, the 3 risk factors identified in our study (history of major bleeding, COVID-19 severity, and use of anticoagulants) can be readily obtained at the time of hospitalization; therefore, they can be applied easily in clinical practice.

In conclusion, our findings demonstrated that among hospitalized patients with COVID-19, the overall incidence of major bleeding was 2.0% during hospitalization, but increased up to 12.3% in patients with severe COVID-19. The independent risk factors for major bleeding were a history of major bleeding, COVID-19 severity, and use of anticoagulant. Bleeding events were associated with a longer duration of hospitalization and higher mortality. Accurate recognition of the risk of bleeding, along with that of thromboembolic events, is warranted to optimize the use of anticoagulants and improve outcomes in patients with COVID-19.

## Data Availability

The data, analytic methods, and study materials will not be made available to other researchers for purposes of reproducing the results or replicating the procedure. However, if the relevant review board or ethics committee approve the data sharing and all investigators of the CLOT-COVID Study give their consent, the deidentified participant data will be shared on a request basis through the principal investigator. Study protocol and statistical analysis plan will also be available. The data will be shared as Excel files via E-mail during the proposed investigation period.

## Abbreviations

COVID-19: Coronavirus disease 2019
VTE: Venous thromboembolism
HbA1c: Hemoglobin A1c
ISTH: International Society of Thrombosis and Hemostasis
APTT: Activated partial thromboplastin time
IQR: Interquartile range
LMWH: Low-molecular-weight heparin
UFH: Unfractionated heparin
DOAC: Direct oral anticoagulant
OR: Odds ratio
CI: Confidence interval

## References

[1] McGonagle D, O'Donnell JS, Sharif K, Emery P, Bridgewood C (2020). Immune mechanisms of pulmonary intravascular coagulopathy in COVID-19 pneumonia. Lancet Rheumatol.

[2] Lorini FL, Di Matteo M, Gritti P, Grazioli L, Benigni A, Zacchetti L (2021). Coagulopathy and COVID-19. Eur Heart J Suppl.

[3] Al-Samkari H, Karp Leaf RS, Dzik WH, Carlson JCT, Fogerty AE, Waheed A (2020). COVID-19 and coagulation: bleeding and thrombotic manifestations of SARS-CoV-2 infection. Blood.

[4] Bikdeli B, Madhavan MV, Jimenez D, Chuich T, Dreyfus I, Driggin E (2020). COVID-19 and thrombotic or thromboembolic disease: Implications for prevention, antithrombotic therapy, and follow-up: JACC State-of-the-Art Review. J Am Coll Cardiol.

[5] Malas MB, Naazie IN, Elsayed N, Mathlouthi A, Marmor R, Clary B (2020). Thromboembolism risk of COVID-19 is high and associated with a higher risk of mortality: A systematic review and meta-analysis. EClinicalMedicine.

[6] Thachil J, Tang N, Gando S, Falanga A, Cattaneo M, Levi M (2020). ISTH interim guidance on recognition and management of coagulopathy in COVID-19. Journal of thrombosis and haemostasis : JTH.

[7] Cuker A, Tseng EK, Nieuwlaat R, Angchaisuksiri P, Blair C, Dane K (2021). American Society of Hematology 2021 guidelines on the use of anticoagulation for thromboprophylaxis in patients with COVID-19. Blood Adv.

[8] Demelo-Rodriguez P, Farfan-Sedano AI, Pedrajas JM, Llamas P, Siguenza P, Jaras MJ (2021). Bleeding risk in hospitalized patients with COVID-19 receiving intermediate- or therapeutic doses of thromboprophylaxis. J Thromb Haemost.

[9] Demelo-Rodriguez P, Galeano-Valle F, Ordieres-Ortega L, Siniscalchi C, Martin Del Pozo M, Fidalgo A, et al. Validation of a prognostic score to identify hospitalized patients with COVID-19 at increased risk for bleeding. Viruses. 2021;13. 10.3390/v13112278. 10.3390/v13112278 PMC8621368 34835085

[10] Al Raizah A, Al Askar A, Shaheen N, Aldosari K, Alnahdi M, Luhanga M (2021). High rate of bleeding and arterial thrombosis in COVID-19: Saudi multicenter study. Thromb J.

[11] Dalager-Pedersen M, Lund LC, Mariager T, Winther R, Hellfritzsch M, Larsen TB (2021). Venous thromboembolism and major bleeding in patients with coronavirus disease 2019 (COVID-19): A nationwide, population-based cohort study. Clin Infect Dis.

[12] Nadkarni GN, Lala A, Bagiella E, Chang HL, Moreno PR, Pujadas E (2020). Anticoagulation, bleeding, mortality, and pathology in hospitalized patients with COVID-19. J Am Coll Cardiol.

[13] Investigators A, Investigators AC-a, Investigators R-C, Lawler PR, Goligher EC, Berger JS (2021). Therapeutic anticoagulation with heparin in noncritically ill patients with Covid-19. N Engl J Med.

[14] Investigators R-C, Investigators AC-a, Investigators A, Goligher EC, Bradbury CA, McVerry BJ (2021). Therapeutic anticoagulation with heparin in critically ill patients with Covid-19. N Engl J Med.

[15] Zellmer S, Hanses F, Muzalyova A, Classen J, Braun G, Piepel C (2021). Gastrointestinal bleeding and endoscopic findings in critically and non-critically ill patients with corona virus disease 2019 (COVID-19): Results from Lean European Open Survey on SARS-CoV-2 (LEOSS) and COKA registries. United European Gastroenterol J.

[16] Yamashita Y, Yachi S, Takeyama M, Nishimoto Y, Tsujino I, Nakamura J (2022). Influence of sex on development of thrombosis in patients with COVID-19: From the CLOT-COVID study. Thromb Res.

[17] Nishimoto Y, Yachi S, Takeyama M, Tsujino I, Nakamura J, Yamamoto N, et al. The current status of thrombosis and anticoagulation therapy in patients with COVID-19 in Japan: From the CLOT-COVID study. J Cardiol. 2022;In Press. 10.1016/j.jjcc.2022.03.015 PMC8979768 35430141

[18] Sakamoto J, Yamashita Y, Morimoto T, Amano H, Takase T, Hiramori S (2019). Cancer-Associated Venous Thromboembolism in the Real World- From the COMMAND VTE Registry. Circ J.

[19] Billett HH, Reyes-Gil M, Szymanski J, Ikemura K, Stahl LR, Lo Y (2020). Anticoagulation in COVID-19: Effect of enoxaparin, heparin, and apixaban on mortality. Thromb Haemost.

[20] Schulman S, Kearon C (2005). Subcommittee on Control of Anticoagulation of the S Standardization Committee of the International Society on T, Haemostasis Definition of major bleeding in clinical investigations of antihemostatic medicinal products in non-surgical patients. J Thromb Haemost.

[21] Ortega-Paz L, Galli M, Capodanno D, Franchi F, Rollini F, Bikdeli B, et al. Safety and efficacy of different prophylactic anticoagulation dosing regimens in critically and non-critically ill patients with COVID-19: A systematic review and meta-analysis of randomized controlled trials. Eur Heart J Cardiovasc Pharmacother. 2021. 10.1093/ehjcvp/pvab070. 10.1093/ehjcvp/pvab070 PMC8499924 34519777

[22] Decousus H, Tapson VF, Bergmann JF, Chong BH, Froehlich JB, Kakkar AK (2011). Factors at admission associated with bleeding risk in medical patients: findings from the IMPROVE investigators. Chest.

[23] Rees EM, Nightingale ES, Jafari Y, Waterlow NR, Clifford S, CA BP (2020). COVID-19 hospitalization duration: a systematic review and data synthesis. BMC Med.

[24] Williamson EJ, Walker AJ, Bhaskaran K, Bacon S, Bates C, Morton CE (2020). Factors associated with COVID-19-related death using OpenSAFELY. Nature.

[25] Li W, Xu Z, Xiang H, Zhang C, Guo Y, Xiong J (2021). Risk factors for systemic and venous thromboembolism, mortality and bleeding risks in 1125 patients with COVID-19: relationship with anticoagulation status. Aging (Albany NY).

[26] Shen L, Qiu L, Liu D, Wang L, Huang H, Ge H (2022). The association of low molecular weight heparin use and in-hospital mortality among patients hospitalized with COVID-19. Cardiovasc Drugs Ther.

[27] Halaby R, Cuker A, Yui J, Matthews A, Ishaaya E, Traxler E (2021). Bleeding risk by intensity of anticoagulation in critically ill patients with COVID-19: A retrospective cohort study. J Thromb Haemost.

[28] Ward A, Sarraju A, Lee D, Bhasin K, Gad S, Beetel R (2022). COVID-19 is associated with higher risk of venous thrombosis, but not arterial thrombosis, compared with influenza: Insights from a large US cohort. PLoS ONE.

[29] Chen J, Hang Y (2021). Characteristics, risk factors and outcomes of gastrointestinal hemorrhage in COVID-19 patients: A meta-analysis. Pak J Med Sci.

[30] Marasco G, Maida M, Morreale GC, Licata M, Renzulli M, Cremon C (2021). Gastrointestinal bleeding in COVID-19 patients: A systematic review with meta-analysis. Can J Gastroenterol Hepatol.

[31] Alkhamis A, Alshamali Y, Alyaqout K, Lari E, Alkhamis MA, Althuwaini S (2021). Prevalence, predictors and outcomes of bleeding events in patients with COVID-19 infection on anticoagulation: Retrospective cohort study. Ann Med Surg (Lond).

[32] Godier A, Clausse D, Meslin S, Bazine M, Lang E, Huche F (2021). Major bleeding complications in critically ill patients with COVID-19 pneumonia. J Thromb Thrombolysis.

[33] Piran S, Schulman S (2019). Treatment of bleeding complications in patients on anticoagulant therapy. Blood.

[34] Lauzier F, Arnold DM, Rabbat C, Heels-Ansdell D, Zarychanski R, Dodek P (2013). Risk factors and impact of major bleeding in critically ill patients receiving heparin thromboprophylaxis. Intensive Care Med.

[35] Mauro A, De Grazia F, Lenti MV, Penagini R, Frego R, Ardizzone S (2021). Upper gastrointestinal bleeding in COVID-19 inpatients: Incidence and management in a multicenter experience from Northern Italy. Clin Res Hepatol Gastroenterol.

[36] Musoke N, Lo KB, Albano J, Peterson E, Bhargav R, Gul F (2020). Anticoagulation and bleeding risk in patients with COVID-19. Thromb Res.

[37] Lawton MT, Alimohammadi E, Bagheri SR, Bostani A, Vaziri S, Karbasforoushan A (2021). Coronavirus disease 2019 (COVID-19) can predispose young to Intracerebral hemorrhage: a retrospective observational study. BMC Neurol.

[38] Kessler C, Stricker H, Demundo D, Elzi L, Monotti R, Bianchi G (2020). Bleeding prevalence in COVID-19 patients receiving intensive antithrombotic prophylaxis. J Thromb Thrombolysis.

[39] Yamashita Y, Maruyama Y, Satokawa H, Nishimoto Y, Tsujino I, Sakashita H (2021). Incidence and clinical features of venous thromboembolism in hospitalized patients with coronavirus disease 2019 (COVID-19) in Japan. Circulation.

[40] Trindade AJ, Izard S, Coppa K, Hirsch JS, Lee C, Satapathy SK (2021). Gastrointestinal bleeding in hospitalized COVID-19 patients: a propensity score matched cohort study. J Intern Med.

[41] Zhao X, Tao M, Chen C, Zhang Y, Fu Y (2021). Clinical features and factors associated with occult gastrointestinal bleeding in COVID-19 patients. Infect Drug Resist.

[42] Al-Samkari H, Gupta S, Leaf RK, Wang W, Rosovsky RP, Brenner SK (2021). Thrombosis, bleeding, and the observational effect of early therapeutic anticoagulation on survival in critically ill patients with cOVID-19. Ann Intern Med.

[43] Guo A, Lu J, Tan H, Kuang Z, Luo Y, Yang T (2021). Risk factors on admission associated with hospital length of stay in patients with COVID-19: a retrospective cohort study. Sci Rep.

